# Physician Gestalt for Anemia Detection in the Emergency Department: A Prospective Study

**DOI:** 10.5811/westjem.48717

**Published:** 2026-01-26

**Authors:** Yun-Chang Chen, Shu-Shien Hsu, Chiat Qiao Liew, Chih-Wei Sung, Chia-Hsin Ko, Chien-Hua Huang, Ming-Tai Cheng, Chu-Lin Tsai

**Affiliations:** * National Taiwan University Hospital Yun-Lin Branch, Department of Emergency Medicine, Yunlin, Taiwan; † National Taiwan University Hospital, Department of Emergency Medicine, Taipei, Taiwan; ‡ National Taiwan University, College of Medicine, Department of Emergency Medicine, Taipei, Taiwan; § National Taiwan University Hospital Hsin-Chu Branch, Department of Emergency Medicine, Hsinchu, Taiwan

## Abstract

**Introduction:**

Anemia is common in the emergency department (ED). Physicians often rely on inspecting conjunctival pallor or other body parts for gestalt estimates. We aimed to evaluate the validity and reliability of physician gestalt for anemia detection and examine the impact of clinical experience and incorporating images of multiple body parts on physician gestalt-based anemia detection.

**Methods:**

Prospective observational study in the ED at an academic medical center between January–November 2023. Using convenience sampling, we included patients ≥ 18 years with recent laboratory hemoglobin (Hgb) measurements. We used a smartphone to capture the images of the patient’s conjunctiva, palm, and fingernails. Five board-certified attending emergency physicians (two junior, two mid-level, and one senior) reviewed the patient images and provided gestalt predictions of Hgb levels and anemia likelihood on a 1–10 scale. Two pairs of physicians evaluated the same set of patient images to assess reliability. Anemia was defined as Hgb < 13.1 grams per deciliter (g/dL) for men and < 11.0 g/dL for women, according to our laboratory standard.

**Results:**

We enrolled a total of 100 patients (mean age 67 years; 45% male). Of these, 59 (59%) had anemia and 41 (41%) did not. The correlation coefficients between physicians’ predicted Hgb levels and actual Hgb levels were only moderate (0.31, 0.41, and 0.40 for junior, mid-level, and senior physicians, respectively; P < .05 for all). Although not statistically significant, the mid-level physicians’ gestalt had the highest area under the receiver operating characteristic curve (0.78), followed by senior- (0.74) and junior physicians (0.72). The impact of incrementally adding images of other body parts to conjunctiva was small (mean changes in anemia likelihood <1 on a 1–10 scale). The agreement on predicted Hgb levels between the paired physicians was high (0.71 for junior physicians, 0.67 for mid-level physicians, P < .001 for both).

**Conclusion:**

Physician gestalt demonstrated moderate validity and moderate-to-high reliability for anemia detection. Adding images other than conjunctiva did not improve the performance of physician gestalt. However, the clinical experience did matter slightly in detecting anemia.

## INTRODUCTION

Anemia is a common medical problem worldwide, affecting about two billion people or one-third of the world’s population.^1^,^2^ The prevalence of anemia in the emergency department (ED) is 28–47%, with a higher prevalence in children, women, and the elderly.^3^,^4^ Acute anemia may be traumatic or due to gastrointestinal (GI) bleeding, while chronic anemia is due to cancer or blood disorders.^5^ Early detection of anemia may facilitate blood preparation for transfusion as needed and may have an impact on patient outcomes.^6^–^8^ For example, receipt of a whole-blood transfusion earlier at any time point within the first 24 hours of ED arrival was associated with improved survival in patients presenting with severe traumatic hemorrhage.^7^ On the contrary, a longer time to red blood cell transfusion was associated with an increased risk of 30-day and in-hospital mortality of patients with GI bleeding.^8^

The gold standard for diagnosing anemia requires blood sampling to determine hemoglobin levels. However, this procedure may be unavailable in resource-limited settings (eg, rural EDs or ED in developing countries without a laboratory)^9^ or may cause delay in ED management. To quickly screen for anemia, clinicians often resort to inspecting conjunctival pallor, although the performance of this method is quite variable.^10^–^12^ Studies in African general populations have demonstrated its effectiveness in identifying severe anemia in children^12^,^13^ and pregnant women.^14^ In the outpatient setting, the presence of conjunctival pallor, without other information suggesting anemia, is reason enough to perform a hemoglobin determination.^15^

The inspection of conjunctival pallor is a simple form of physician gestalt, defined as the formulation of first impressions through clinical intuition, knowledge, and experience.^16^,^17^ The term “gestalt,” derived from German, means “pattern” or “shape.” Rooted in psychology, it is commonly associated with the idea that “the whole is greater than the sum of its parts.”^18^ Early gestalt research often emphasized visual inspection, as it was straightforward.^18^

To the best of our knowledge, there has been only one ED study (in Africa) addressing the validity of physician gestalt for determining the presence and severity of anemia.^19^ In that study, physicians provided a gestalt estimate of the presence and severity of anemia considering the pallor of the conjunctiva, nail beds, lips, oral mucosa, and palmar creases. These tissues have fewer or no melanocytes^20^ and, thus, are less affected by various skin colors (ie, Fitzpatrick skin types).^21^ The study reported a moderate correlation (Kendall’s tau of 0.63) between physician gestalt and the measured Hgb categorizations.^19^ Whether the results can be generalized to other ED populations (eg, non-African populations with less color contrast between skin color and melanocyte-free regions) remains unclear.

To better understand the role of gestalt-based anemia detection, we aimed to 1) evaluate the validity and reliability of physician gestalt, and 2) study the impact of clinical experience and incorporating multiple body parts images of tissue and mucous membranes. We hypothesized that physician gestalt for anemia detection was valid and reliable and improved with clinical experience and additional imaging.

**Population Health Research Capsule**

What do we already know about this issue?

*Clinicians often visually assess conjunctival pallor to diagnose anemia, but its diagnostic validity and reliability remain uncertain*.

What was the research question?

*How valid and reliable is physician gestalt for anemia detection in emergency department (ED) patients using body part images?*

What was the major finding of the study?

*Gestalt had moderate validity (correlation = 0.31–0.41; P <.05) and high reliability (correlation = 0.67–0.71, P < .001)*.

How does this improve population health?

*Identifying the limits of visual anemia assessment helps guide training and promotes computer vision-assisted rapid anemia screening in the ED*.

## METHODS

### Study Design, Setting, and Population

This was a prospective observational study conducted in the ED of the National Taiwan University Hospital (NTUH) from January–November 2023. The NTUH is a tertiary academic medical center with approximately 2,400 beds and 100,000 ED visits annually. Patients presenting to the ED were prospectively enrolled by trained research personnel following a standardized protocol. By using convenience sampling, patients > 18 years of age with recent laboratory hemoglobin (Hgb) measurement (within 24 hours) before enrollment were included. The exclusion criteria were as follows: 1) active eye diseases; 2) hypoxemia (SpO_2_ < 90%) at triage; 3) inability or unwillingness to provide written consent; 4) receiving transfusion between the time of Hgb measurement and the time photos were taken.

Using an iPhone 13 (Apple Inc., Cupertino, CA), we captured images of the patient’s conjunctiva, palm, and fingernails under ambient lighting. A trained research assistant who was blinded to the patients’ Hgb levels captured all images. Prior to imaging, the assistant tapped the screen to focus on the tissue of interest. To ensure consistent images, each image was taken at approximately 20 cm from the patient’s conjunctiva or palm/fingernails. Patients were asked to gently curl their fingers inward with palms facing upward to avoid blanching caused by excessive extension or flexion or to gently place their fingers on a white clipboard. Photographs were taken without flash for conjunctiva to avoid bright conjunctival reflections and with flash for palm/fingernails.

Each image was visually assessed by a research associate for quality assurance. In cases of potential quality issues, the image was referred to the principal investigator to determine whether it should be excluded. Basic ED data were collected, including demographics, triage level, mode of arrival, structured chief complaints, ED disposition, and ED length of stay. Data were directly extracted via structured items in the Taiwan Triage and Acuity Scale (TTAS) embedded in our electronic health records system. The TTAS, a computerized triage software adapted from the Canadian Triage and Acuity Scale (CTAS), has been used for ED triage in Taiwan since 2010.^17^

This study was approved by the NTUH Institutional Review Board, and informed consent was obtained from all participants or their surrogates.

### Physician Gestalt for Anemia Detection

**Figure 1:**
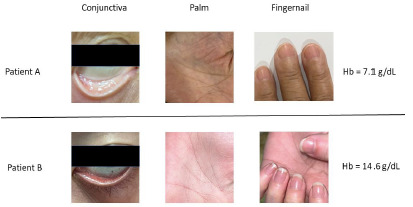
Photographs of tissue/membranes studied in a patient with anemia (Patient A) and without anemia (Patient B). The corresponding hemoglobin levels are 7.1 grams per deciliter (g/dL) and 14.6 g/dL, respectively. g/dL, grams per deciliter.

We recruited five emergency medicine board-certified attending physicians with superior clinical skills via voluntary participation and introduced them to the study through research meetings. We divided the physicians into three groups based on their years of attending experience: two junior (attending physician year 3 [APY-3]), two mid-level (APY-7), and one senior (AP16) physician. Only images of patients and patients’ age and sex were provided to the reviewers. All physicians were blinded to the study hypothesis and test results. Two sets of representative images along with the Hgb levels are shown in Figure 1.

Physician reviewers were asked to evaluate the likelihood of anemia based on a Likert scale from 1–10, with higher values indicating an increased likelihood of anemia. This scale can be thought of as the rating of the physician’s pre-test probability of anemia based on the patient’s information (image, age, and sex). This approach would better quantify the incremental effects of adding additional tissue and membrane images and facilitate the receiver operating characteristic (ROC) curve analysis. They were also asked to provide integer-based predicted Hgb levels. Conjunctiva images were the primary tissues used to assess anemia. To assess the impact of adding images from other tissues (ie, palm and fingernails) on anemia detection, one physician from the junior, mid-level, and senior groups re-evaluated the anemia likelihood in an incremental manner of adding images of palm and fingernails (n = 100). To assess the reliability of physician gestalt, a pair of physicians from the same experience group (junior and mid-level) evaluated the same first half set of conjunctiva images (n = 50).

### Outcome Measure

Venous Hgb levels in the study were measured by the EDlaboratory, which is considered the gold standard. The normal Hgb level for the hospital’s laboratory was 13.1–17.2 grams per deciliter (g/dL) for men and 11.0–15.2 g/dL for women. Any levels below these cutoff points were considered indicative of anemia (dichotomous).

### Statistical Analysis

Summary statistics are presented as proportions (with 95% confidence intervals), means (with standard deviations), or medians (with interquartile ranges). The overview of the analysis plan is shown in the supplementary figure. We assessed the predictive accuracy (validity) of physician gestalt by calculating Spearman correlation coefficients between physicians’ predicted Hgb levels (based on conjunctiva images alone) and actual Hgb levels measured by the lab. We also evaluated the discriminatory ability of physicians’ predictions of likelihood of anemia using the area under the receiver operating characteristic (ROC) curve (AUC) against the true dichotomized anemia status.

The AUC analysis was further stratified by physician experience group and tissue combinations. The DeLong test was used for the comparison between AUCs. A subgroup AUC analysis of GI bleeding (eg, acute anemia) was performed. A sensitivity analysis of an alternative cutoff point for anemia (Hgb < 10 g/dL) regardless of sex was performed to assess the impact of different anemia criteria. Another sensitivity analysis removed mild anemia cases (anemic patients with Hgb > 10 g/dL) from the analysis to see whether physician gestalt’s discriminatory ability improved.

We calculated the mean changes in anemia likelihood to assess the impact of incrementally adding tissue/membrane other than the conjunctiva. For the reliability analysis, the kappa statistic was used to measure the categorical agreement between the anemia likelihood (> 5 on a scale of −10) provided by the pairs of physicians. We also used the Spearman correlation coefficients and Bland-Altman plots to evaluate the agreement between the paired physicians’ predictions of Hgb levels in junior and mid-level physician groups.

All analyses were performed using Stata 16.0 software (StataCorp, College Station, TX). All *P*-values are two-sided, with *P* < .05 considered statistically significant.

## RESULTS

**Figure 2:**
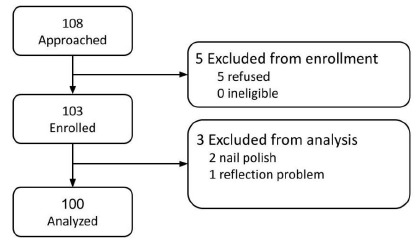
Participant flow diagram

Figure 2 illustrates the participant flow. A total of 108 individuals were approached for the study, with 103 enrolled and five excluded due to refusal. Of the enrolled, three were later excluded from the analysis: two for wearing nail polish and one due to reflection issues in photos taken. A total of 100 participants were included in the final analysis.

**Figure 3:**
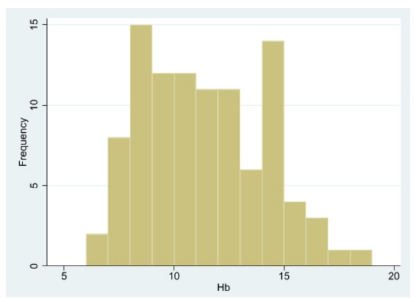
The distribution of hemoglobin levels in this study.

The baseline characteristics of the enrolled patients are shown in Table 1. The average age of the participants was 67.3 years, with females comprising 55% (n=55) of the cohort. A small proportion (7%) of patients arrived at the ED by ambulance. The most common chief complaints were fever (17%), followed by abdominal pain (12%) and shortness of breath (9%). Triage assessments indicated that most patients (84%) were classified as urgent (Level 3). Anemia was present in 59% of the patients, with an average Hgb level of 11.3 g/dL. The distribution of Hgb is shown in Figure 3. Of them, 37 patients had an Hgb level < 10.0 g/dL; only two had an Hgb level < 7.0 g/dL; 11% of patients received a transfusion of packed red blood cells. Approximately 59% of the patients were hospitalized after their ED visit. The median ED stay was 45.4 hours for the entire cohort.

Regarding the predictive validity, the correlation coefficients between physicians’ predicted Hgb levels (based on conjunctiva images) and actual Hgb levels were only moderate. The correlation coefficients were 0.31 (95% CI, 0.13–0.48), 0.41 (95% CI, 0.24–0.57), and 0.40 (95% CI, 0.22–0.56) for junior, mid-level, and senior attending physicians, respectively (*P* < .05 for all).

**Figure 4:**
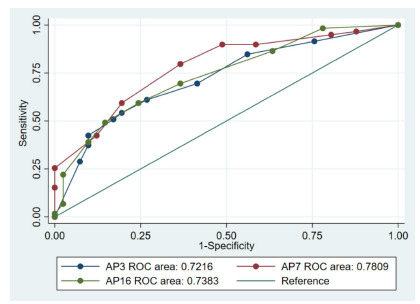
Receiver operating characteristic curves for anemia detection with conjunctiva images alone across three physician experience groups. AP, attending physician years in practice; *ROC*, receiver operating characteristic.

Table 2 shows the discriminatory ability of physician gestalt for anemia detection stratified by physician experience and tissue/membrane combinations. Based on conjunctiva images alone, the mid-level physician’s gestalt had the highest discriminatory power, with the highest AUC (0.78). The senior physician’s gestalt had the second-highest AUC (0.74), followed by the junior physician’s gestalt (0.72). Adding images for other tissue/membranes appeared to have a different impact among these three experience groups. The addition of images improved the AUC for the junior physician (from 0.72 to 0.81) but not for mid-level and senior physicians. The ROC curves in Figure 4 visually corroborated the numerical AUC findings. The AUC appeared to be highest for the mid-level physician, followed by the senior and junior physicians; however, the differences in AUCs were not statistically significant (*P* = .35).

The impact of incrementally adding tissue/membranes other than the conjunctiva on the assessment of anemia likelihood is shown in Supplementary Table 1. The mean changes in anemia likelihood appeared to be small, with changes < one (on a scale of 1–10) across all physician experience groups.

**Figure 5:**
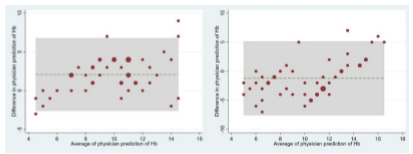
The Bland-Altman plots of the agreement of hemoglobin levels by paired physicians of the same experience group (junior physicians on the left panel; mid-level physicians on the right panel). The green dashed line represents the mean difference between the two physician groups’ predicted levels. The shaded box is bounded by the statistical limits of agreement, which are defined as the mean difference ± 1.96 SD of differences. The sizes of circles are proportional to the number of observations.

The agreement between predicted Hgb levels by the paired physicians from the same experience group is shown in Figure 5. For both panels, the analysis demonstrated high agreement, as most observations were confined within the shaded box (the statistical limits of agreement). Similarly, the correlation coefficients between were high for the junior physicians (0.71, *P* < .001) and the mid-level physicians (0.67, *P* < .001). The kappa statistic showed a strong agreement (0.80) between the junior physicians and a fair agreement (0.37) between the mid-level physicians.

The Hgb levels for the subgroup of GI bleeding (n=14) ranged from 6.4–15.5 g/dL. The AUC analysis of this subgroup (eg, acute anemia) was variable, ranging from 0.58–1.00 across the physician experience groups. A sensitivity analysis of an alternative cutoff point for anemia (Hgb < 10 g/dL), regardless of sex, showed largely acceptable AUCs across the physician experience groups (Supplementary Table 2). Removing mild anemia cases (anemic patients with Hgb > 10 g/dL) from analysis improved physician gestalt’s discriminatory ability (increased AUCs), as shown in Supplementary Table 3. The test characteristics of mid-level physician gestalt for anemia detection (anemia likelihood) via conjunctiva are shown in Supplementary Table 4.

## DISCUSSION

In this prospective ED observational study, five attending emergency physicians provided gestalt estimates of Hgb levels and anemia likelihood based on 100 images from each of the conjunctiva, palm, and fingernails. Three major themes emerged from the analysis: 1) the predictive validity was only moderate, while the discriminatory validity was acceptable and improved with clinical experience; 2) the incremental discriminatory value of additional tissue/membrane images was variable, with minimal changes in anemia likelihood; and 3) the agreement on predicted Hgb levels between the paired physicians was moderate to high.

The moderate correlation between physicians’ predicted Hgb levels and the actual Hgb levels was disappointing but not surprising. Limited “image samples” are acquired during emergency medicine residency training, and often these samples’ labels (Hgb levels) are not remembered in the long term. This moderate correlation (or accuracy) has been reported in previous studies.^10^,^11^ In contrast, physicians seemed to be able to rank the likelihood of anemia well, as supported by acceptable discriminatory validity (AUCs of 0.7–0.8). It seems that by retrieving information from each physician’s “image bank,” the physician can still sort out and differentiate anemia from non-anemia.

In addition, clinical experience appears to contribute to slightly higher discriminatory performance. In our study, the mid-level physician’s gestalt had the highest AUC, outperforming the junior and senior physicians, although the differences were not statistically significant. Similar “experience effects” have been observed in physician gestalt for predicting pulmonary embolism and acute appendicitis, where there was a trend toward increasing accuracy with increasing experience.^22^,^23^ In our study, the slightly decreased AUC in the senior physician might result from less frequent patient care and more administrative/research responsibility in senior physicians, resulting in less sharp clinical skills. This “middle-age-performs-best” phenomenon has also been observed in surgical fields. For example, in a study of the performance of thyroid surgeons, which found higher complication rates for senior surgeons, the authors speculated that the decline in performance in senior surgeons might have been due to physiologic factors and/or not keeping up with new techniques.^24^

We also found that the incremental value of additional tissue/membrane images was variable, with minimal changes in anemia likelihood. Previous studies have shown that areas with fewer or no melanocytes, such as conjunctiva, tongue, lips, fingernails, and palm, are more suitable for observing anemia^10^,^11^,^25^; however, there is no consensus on which tissue or membrane is most useful. A study in India found that tongue pallor outperformed other pallor sites (conjunctiva, palm, and nailbed) and was the best discriminator of anemia at hemoglobin thresholds of 7 and 9 g/dL.^11^ Another study in Pakistan showed that all pallor sites of the conjunctiva, nailbed, and palm were equally useful for detecting severe anemia.^10^ To our knowledge, our study is the first to investigate the incremental values of tissue/membrane images in anemia detection. The results, however, were quite variable, probably due to the variable experience of inspecting palms and fingernails among physicians.

Regarding reliability, physicians of the same experience levels demonstrated moderate to high agreement in predicting Hgb levels. Although some studies have shown significant differences in consistency among observers,^10^,^11^ other studies have yielding results similar to ours.^19^,^26^,^27^ The ones showing more consistency were single-centered studies, including ours, with similar training among physicians. Given the moderate validity, it is also possible that physicians made the same mistakes because of the atypical presentation of conjunctiva images. Subtle conjunctiva vascularity missed by the human eyes may be captured by machine or deep learning techniques, a promising non-invasive approach to detecting anemia either in the ED or at home.^28^–^31^

Researchers in future studies may consider including more critically ill patients triaged at level 1 or 2 or with Hgb levels <n7 g/dL, as early detection of anemia via physician gestalt may have more impact on the outcomes of these patients requiring emergent blood. Our study included mostly triage level 3 patients who were often boarders in the ED awaiting inpatient beds, as reflected by their prolonged ED length of stay. Further research may also consider face-to-face assessments to generate physician gestalt estimates of anemia, not just photos of specific tissue or membrane. A variety of physical clues could contribute to gestalt estimates of anemia, such as facial expression and verbal communication, prior to blood work. A previous study has shown decreased facial expression variability in patients with serious cardiopulmonary disease in the ED.^32^ The interaction of the physician and the patient may provide more information for physician gestalt, as well as more distracting factors. The overall effect of added information on the accuracy of physician gestalt of anemia detection would require further study.

## LIMITATIONS

The study has several limitations. First, the images were collected in the ED under ambient lighting, and some variations in lighting may have affected the interpretation. Second, physicians were provided with patients’ images, age, and sex for estimation. The physicians might have performed differently if they had seen and examined the patients themselves. Third, because our study was comprised of a convenience sample rather than a consecutive ED sample, this approach may have introduced some bias with respect to the spectrum of anemic patients in the ED. Specifically, this study did not include critically ill patients requiring emergent blood. Finally, we excluded patients < 18 years of age, because their normal Hgb levels are different from those of adults. Therefore, our results would not be generalizable to children.

## CONCLUSION

In this prospective ED study, physician gestalt demonstrated moderate validity and moderate-to-high reliability for anemia detection. Adding images other than conjunctiva did not seem to improve the performance of physician gestalt. However, the clinical experience did matter slightly in detecting anemia. To improve gestalt detection of anemia, physicians need more training (or experience) to build their own bank of tissue and membrane images with Hgb labels. Similar learning principles can be applied to machines using computer vision algorithms for anemia detection, which may aid physicians with limited experience in the ED and expedite blood preparation, potentially improving patient care.

## Supplementary Information











## Figures and Tables

**Table 1:** Baseline clinical characteristics of emergency department patients.

Variable	N = 100
Age, mean (SD), yr	67.3 (16.6)
Female sex, n (%)	55 (55.0)
Arrival by ambulance, n (%)	7 (7.0)
Most common chief complaint, n (%)	
Fever	17 (17.0)
Abdominal pain	12 (12.0)
Shortness of breath	9 (9.0)
Triage level, n (%)	
1 (Resuscitation)	1 (1.0)
2 (Emergent)	15 (15.0)
3 (Urgent)	84 (84.0)
4 (Less urgent)	0 (0)
5 (Non-urgent)	0 (0)
Anemia, n (%)	59 (59.0)
Hgb level, mean (SD), g/dL	11.3 (2.8)
Transfusion of packed red blood cells, n (%)	11 (11.0)
Hospital admission, n (%)	59 (59.0)
ED length of stay, median (IQR), hr	45.4 (26.3–71.7)

*ED*, emergency department; g/dL, grams per deciliter.

**Table 2:** The area under the receiving operating curve by attending physician experience.

	Junior (APY3)	Mid-level (APY7)	Senior (APY16)
Conjunctiva	0.7216	0.7809	0.7383
Conjunctiva + palm	0.7621	0.7187	0.7354
Conjunctiva + palm + fingernails	0.8138	0.7857	0.7199

*APY*, attending physician’s years of experience.
