# Supplementary material for: Physician Gestalt for Anemia Detection in the Emergency Department: A Prospective Study
Source: West J Emerg Med. 2026 Jan 26;27(2):337–44. doi: 10.5811/westjem.48717 (PMC13016077; doi:10.5811/westjem.48717)
Supplement: Supplementary file 1 [file wjem-27-337-s001.docx]

**Online Supplementary Figure.** The overview of the analysis plan.


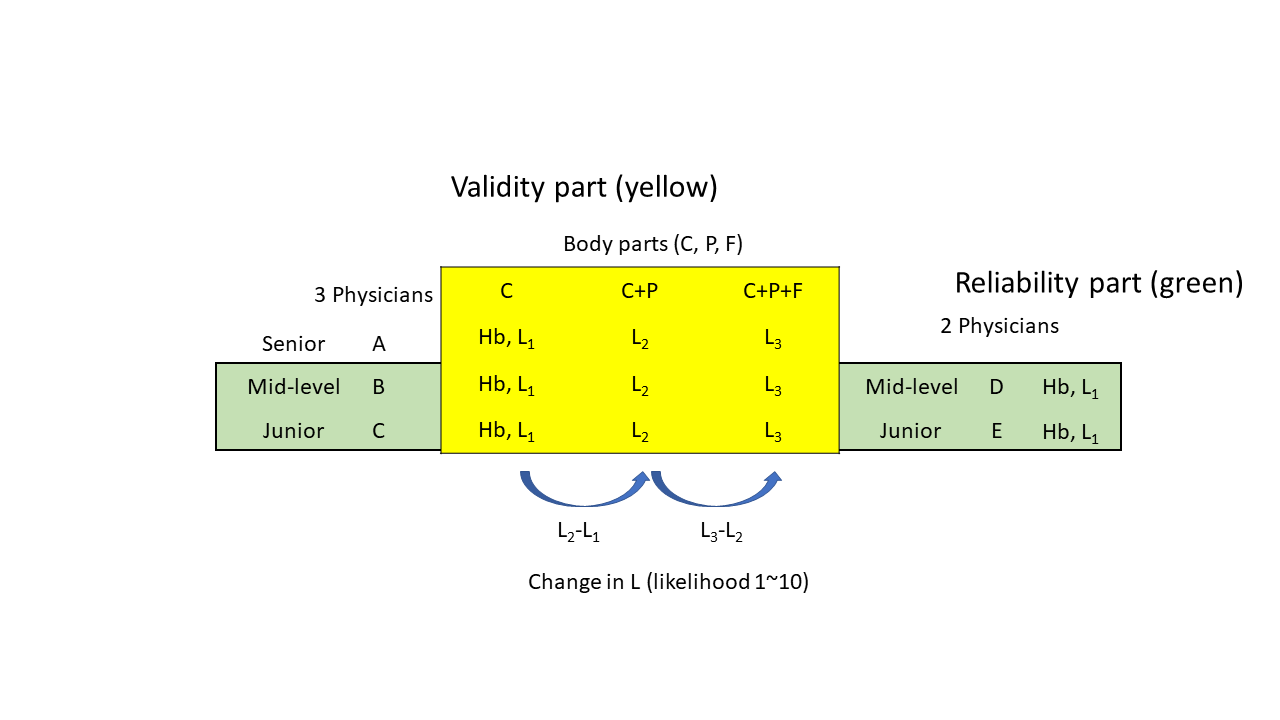


Abbreviations: C = conjunctiva; P = palm; F = fingernail; L = likelihood of anemia.
