# Supplementary material for: Physician Gestalt for Anemia Detection in the Emergency Department: A Prospective Study
Source: West J Emerg Med. 2026 Jan 26;27(2):337–44. doi: 10.5811/westjem.48717 (PMC13016077; doi:10.5811/westjem.48717)
Supplement: Supplementary file 2 [file wjem-27-337-s002.docx]

**Supplementary Table 1**. Mean changes in anemia likelihood after sequentially reviewing additional images.

|  | Base: conjunctiva | Base: conjunctiva + palm |
| --- | --- | --- |
|  | Mean change in anemia likelihood after reviewing ***additional palm images*** | Mean change in anemia likelihood after reviewing ***additional fingernail images*** |
| Junior (APY3) | -0.21 | 0.26 |
| Mid-level (APY7) | -0.26 | -0.11 |
| Senior (APY16) | -0.38 | -0.21 |

Abbreviation: APY = attending physician’s year of experience.
