# Supplementary material for: Physician Gestalt for Anemia Detection in the Emergency Department: A Prospective Study
Source: West J Emerg Med. 2026 Jan 26;27(2):337–44. doi: 10.5811/westjem.48717 (PMC13016077; doi:10.5811/westjem.48717)
Supplement: Supplementary file 3 [file wjem-27-337-s003.docx]

**Supplementary Table 2**. The area under the receiving operating curve by attending physician experience using hemoglobin <10 g/dL as the cutoff point for anemia.

|  | Junior (APY3) | Mid-level (APY7) | Senior (APY16) |
| --- | --- | --- | --- |
| Conjunctiva | 0.6665 | 0.7372 | 0.7458 |
| Conjunctiva + palm | 0.7396 | 0.7033 | 0.7383 |
| Conjunctiva + palm + fingernails | 0.7482 | 0.7121 | 0.7126 |

Abbreviation: APY = attending physician’s year of experience.
