# Supplementary material for: Physician Gestalt for Anemia Detection in the Emergency Department: A Prospective Study
Source: West J Emerg Med. 2026 Jan 26;27(2):337–44. doi: 10.5811/westjem.48717 (PMC13016077; doi:10.5811/westjem.48717)
Supplement: Supplementary file 4 [file wjem-27-337-s004.docx]

**Supplementary Table 3**. The area under the receiving operating curve by attending physician experience after excluding mild anemia cases (anemic patients with Hb>10 g/dL).

|  | Junior (APY3) | Mid-level (APY7) | Senior (APY16) |
| --- | --- | --- | --- |
| Conjunctiva | 0.7376 | 0.8088 | 0.7894 |
| Conjunctiva + palm | 0.8078 | 0.7561 | 0.7914 |
| Conjunctiva + palm + fingernails | 0.8385 | 0.8022 | 0.7564 |

Abbreviation: APY = attending physician’s year of experience.
