# Supplementary material for: Physician Gestalt for Anemia Detection in the Emergency Department: A Prospective Study
Source: West J Emerg Med. 2026 Jan 26;27(2):337–44. doi: 10.5811/westjem.48717 (PMC13016077; doi:10.5811/westjem.48717)
Supplement: Supplementary file 5 [file wjem-27-337-s005.docx]

**Supplementary Table 4**. Test characteristics of mid-level physician gestalt for anemia detection (anemia likelihood) via conjunctiva.

| **Cut point** | **Sensitivity, %** | **Specificity, %** | **LR+** | **LR-** |
| --- | --- | --- | --- | --- |
| ≥ 1 | 100 | 0 | 1.0 |  |
| ≥ 2 | 97 | 12 | 1.1 | 0.3 |
| ≥ 3 | 95 | 20 | 1.2 | 0.3 |
| ≥ 4 | 90 | 41 | 1.5 | 0.2 |
| ≥ 5 | 90 | 51 | 1.8 | 0.2 |
| ≥ 6 | 80 | 63 | 2.2 | 0.3 |
| ≥ 7 | 59 | 80 | 3.0 | 0.5 |
| ≥ 8 | 42 | 88 | 3.5 | 0.7 |
| ≥ 9 | 15 | 100 |  | 0.7 |
| ≥ 10 | 2 | 100 |  | 0.8 |
